# Menarche delay and menstrual irregularities persist in adolescents with type 1 diabetes

**DOI:** 10.1186/1477-7827-9-61

**Published:** 2011-05-06

**Authors:** Bahareh M Schweiger, Janet K Snell-Bergeon, Rossana Roman, Kim McFann, Georgeanna J Klingensmith

**Affiliations:** 1 Barbara Davis Center for Childhood Diabetes, University of Colorado Denver, The Children's Hospital Aurora, Colorado, USA

## Abstract

**Background:**

Menarche delay has been reported in adolescent females with type 1 diabetes (T1DM), perhaps due to poor glycemic control. We sought to compare age at menarche between adolescent females with T1DM and national data, and to identify factors associated with delayed menarche and menstrual irregularity in T1DM.

**Methods:**

This was a cross-sectional study and females ages 12- 24 years (n = 228) with at least one menstrual period were recruited during their outpatient diabetes clinic appointment. The National Health and Nutrition Examination Survey (NHANES) 2001-2006 data (n = 3690) for females 12-24 years were used as a control group.

**Results:**

Age at menarche was later in adolescent females with T1DM diagnosed prior to menarche (12.81 +/- 0.09 years) (mean+/- SE) (n = 185) than for adolescent females diagnosed after menarche (12.17 0.19 years, *p = *0.0015) (n = 43). Average age of menarche in NHANES was 12.27 +/- 0.038 years, which was significantly earlier than adolescent females with T1DM prior to menarche (*p *< 0.0001) and similar to adolescent females diagnosed after menarche (*p *= 0.77). Older age at menarche was negatively correlated with BMI z-score (r = -0.23 *p = *0.0029) but not hemoglobin A1c (A1c) at menarche (r = 0.01, *p *= 0.91). Among 181 adolescent females who were at least 2 years post menarche, 63 (35%) reported usually or always irregular cycles.

**Conclusion:**

Adolescent females with T1DM had a later onset of menarche than both adolescent females who developed T1DM after menarche and NHANES data. Menarche age was negatively associated with BMI z-score, but not A1c. Despite improved treatment in recent decades, menarche delay and high prevalence of menstrual irregularity is still observed among adolescent females with T1DM.

## Background

The delay in menstruation in females with type 1 diabetes (T1DM) was appreciated well before the more recent improvement and advances in diabetes treatment introduced by the Diabetes Control and Complications Trial DCCT [1-3]. As a result of the DCCT trial [4], intensive treatment has become the standard of care. However, little research has been done post DCCT to see the effect of improved glycemic control on menarchal timing and menstrual regularity in adolescent females with T1DM, and recent data are limited and often conflicting. Some studies show that menarche is still delayed [5-9], but others have found no delay [10].

Most studies have found that there is a delay in the age of menarche if the onset of diabetes occurs before 10 or 11 years of age or near the onset of puberty [11-15] and that this delay increases with poor glycemic control [16]. However, one study did not find any association between age at menarche and age of onset of T1DM [7]. Another study conducted a retrospective evaluation of 62 adolescent male and females followed longitudinally from the onset of T1DM until final height was reached and found that pubertal development and progression occurred independent of the age at onset of T1DM and glycemic control [17].

It is important to better understand the relationship between T1DM and onset of menarche because menstrual delay can be associated with decreased bone mineral density [18,19] and irregular menses [15,20-24]. Also, women with T1DM have been reported to have earlier natural menopause, fewer pregnancies, more stillbirths [9] and a higher frequency of hyperandrogenism and polycystic ovarian syndrome [25,26] than women without diabetes. Furthermore, women with T1DM are more likely to have 21- hydroxylase autoantibodies (21-OH antibodies), a marker of adrenal autoimmunity, which also are associated with premature ovarian failure [27].

We investigated the effect of type 1 diabetes (T1DM) on age at menarche and menstrual history and sought to identify factors associated with menarche and irregularity in menses. We evaluated factors including A1c, BMI z-score, presence of 21-OH antibodies, episodes of severe hypoglycemia, and episodes of diabetic ketoacidosis (DKA) since these factors have an impact on diabetes management and metabolic outcomes. We sought to identify their potential role in delayed menarche and menstrual irregularity in adolescents with T1D. Additionally, since level of regular physical activity can affect menstrual regularity [28], we also assessed level of physical activity in study subjects.

## Methods

Subjects with T1DM were recruited from the Barbara Davis Center for Childhood Diabetes (BDC). The recruiter was blinded to the presence or absence of menarche prior to approaching the subjects for participation. Females of all ethnicities and ages who had at least one menstrual period and received their care in the pediatric department of the BDC were enrolled in the study. Subjects with a preexisting diagnosis of Hashimoto's thyroiditis were included if their initial diagnosis was made by close surveillance and if they had a longstanding history of being clinically and biochemically euthyroid. Subjects with celiac disease and any other chronic disease in addition to TIDM were excluded from the study. A total of 233 subjects between the ages of 12 and 24 years of age were enrolled in the study between November 2008 and May 2009. Of the 233 subjects with T1DM, complete data on age at menarche and age at T1DM diagnosis were available for 228. The age of diagnosis of T1DM was not available in four subjects and two subjects, both 15 years of age, had not yet started their menstrual periods. These six subjects were excluded from the analysis. There were 23 subjects with stable, treated hypothyroidism included in the analysis. Characteristics of the study participants are shown in Table 1. Of the 228 females with T1DM, 185 participants had diabetes onset prior to menarche and 43 were diagnosed with T1DM after menarche.

**Table 1:** Demographics of subjects with T1DM

	Participants diagnosed with T1DM before menarche (n = 185)	Participants with T1DM diagnosed after menarche (n = 43)
Age at visit (years)	15.8 ± 0.2	16.5 ± 0.3
Duration (years)	7.7 ± 0.4**	2.5 ± 0.312.17 ± 0.19
Age at Menarche (years)	12.81 ± 0.09*	
A1c (%)	9.4 ± 0.14	8.8 ± 0.32
Insulin dose (units/kg body weight/day)	1.02 ± 0.02**	0.0.76 ± 0.05
BMI (kg/m2)	23.8 ± 0.4	23.9 ± 1.0
BMI z score	0.74 ± 0.05	0.56 ± 0.14
Overweight (≥ 85th percentile for age) (%)	32	35
**Total physical activity**		
Number of days of physical activity over last 7 days that the subject was physically active for at least 60 minutes per day	2.6 ± 0.4	2.6 ± 0.2
In a typical week the number of days that they are physically active for at least 60 minutes per day	3.0 ± 0.3	3.2 ± 0.2
Race/Ethnicity (%)		
White	82	79
Hispanic	8	5
Other	0	6
**Breast tanner staging (%)**		
3	2	3
4	12	28
5	86	69
**Pubic tanner staging**		
3	5	4
4	9	24
5	86	72
Irregular menstrual periods (%)	34	36

Data are presented as mean ± SE or as % (n)

* *p *< 0.05, ** *p *< 0.001

Study approval was obtained from the institutional review board at the University of Colorado Denver and participants provided written informed consent and assent, if appropriate, at enrollment.

The National Heath and Nutrition Examination Surveys (NHANES) 2001-06 reproductive health survey was used as a population-representative comparative control sample of women. The survey is designed to assess the health and nutritional status of the non-institutionalized civilian US population using a complex multi-stage, stratified, clustered sampling design [29]. The 2001-2006 time period was specifically selected as it is the most recent set of data available and the time period is similar to the time of onset of menarche for our subjects. NHANES data are available in 2-yr sets. We selected the three latest sets of data in order to have enough power to analyze the groups by ethnicity.

### Data collection

The clinical diabetes type assigned by the health care provider was obtained from the medical records and categorized as T1DM if the provider assignment was type 1, type 1a or type 1b diabetes [30]. Height, weight, and A1c were measured. A1c was measured on a DCA 2000 (Bayer Diabetes Care, Tarrytown, NY). The reference value for healthy persons is 4-6%. Weight was measured in kg using a Detecto (Webb City, MO) scale. Height was measured in centimeters using a Holtain Limited Stadiometer (Crosswell, UK). Physical examination including Tanner staging was performed by a pediatric endocrinologist. Body mass index (BMI) was calculated as kg/m^2^, and BMI z-score was determined using age and sex specific BMI percentiles from the Centers for Disease Control and Prevention.

Demographic data, including race and ethnicity, was collected by self-report. Physical activity was obtained using the Moderate to Vigorous Physical Activity Screening Measure (MVPA) and was categorized as the number of days subjects had accumulated 60 minutes of MVPA during the past 7 days and for a typical week [31]. Age of the first menstrual period and menstrual cycle pattern and length was obtained by self-report using a questionnaire adapted from the NHANES reproductive health questionnaire. Questions regarding cycle pattern and length were based on times when the subject was not taking hormone therapy (oral contraceptives, depo provera, norplant, etc). Subjects were asked to recall their age at the time of their first menstrual period. Probing questions were asked to help the subjects if they had difficulty remembering the exact age, such as remembering the season, grade or proximity to a birthday [32]. An Insulin Record Questionnaire was used to collect insulin regimen and dose. Insulin dose was analyzed as average units of insulin used per kilogram of weight per day.

A retrospective review of the chart was performed to obtain the A1c and BMI within 3 months of the age of menarche on the 185 subjects diagnosed with T1DM before menarche. As an additional measure of glucose control and variability, data were also obtained through retrospective review of the chart on the number of episodes of diabetic ketoacidosis (DKA), defined as ph < 7.3 and serum bicarbonate < 15, and severe hypoglycemia (defined as documented blood glucose < 50 mg/dl and requiring assistance of another person or associated with loss of consciousness or seizure like activity requiring treatment with glucagon or IV dextrose) within the two years prior to menarche. In addition, 21 OH antibodies are routinely measured on all patients with TIDM receiving their care at BDC and were collected as part of the retrospective chart review.

## Results

### Statistical analysis

An independent samples *t-*test was used to test the difference in age at menarche between study participants who developed T1DM before menarche and those who developed T1DM after menarche. Due to the complex sampling strategy used for NHANES 2001-2006, sample weights were used in PROC SURVEYMEANS (SAS version 9.2, SAS Institute, Cary, NC) to obtain sample estimates and confidence intervals. A one sample Z-test was used to compare the study sample to the NHANES data. An independent samples *t-*test was used to test the difference in age of menarche between study participants who had 21-hydroxylase autoantibodies compared to those without, and to compare age of menarche among study participants with a history of diabetic ketoacidosis (DKA) or severe hypoglycemia compared to those without. A Chi-square test was used to compare a history of menstrual irregularity between study participants with and without a history of DKA, and between adolescent females diagnosed with T1DM pre- vs. post-menarche.

Linear regression analysis was used to examine relationships of age at menarche with clinical factors, including body mass index (BMI) and A1c at the time of menarche, and to examine age at menarche between study participants who developed T1DM before menarche vs. study participants who developed T1DM after menarche, adjusting for age at visit.

### Average age of menarche in adolescents with T1DM

Characteristics of study participants with T1DM are shown in Table 1, by diagnosis of T1DM before vs. after menarche. There was no difference in age at visit, A1c, BMI, BMI z-score, prevalence of overweight, race/ethnicity, Tanner Stage, or menstrual irregularity between these groups. Adolescent females diagnosed pre-menarche had a longer duration of diabetes and a higher daily insulin dose per kg than the group diagnosed with T1DM after menarche.

**Figure 1:**
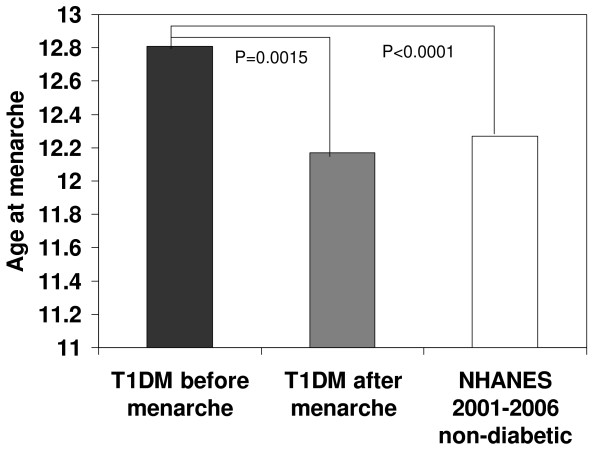
**T1DM before and after menarche versus NHANES**. T1DM before menarche versus NHANES without T1DM, *P *< 0.0001. T1DM after menarche versus NHANES without T1DM, *P *= 0.7676.

The overall mean age of menarche among females with T1DM was 12.69 ± 0.08 years. Adolescent females who developed diabetes before menarche had an average age of menarche of 12.81 ± 0.09 years compared to 12.17 ± 0.19 years, p = 0.0015 (Figure 1) in those who developed diabetes after menarche. In multivariable linear regression analysis further adjusting for age at visit, the average age of menarche among adolescent females diagnosed with T1DM before menarche remained significantly older than the age of menarche among adolescent females diagnosed with T1DM after menarche (least square means 12.82 ± 0.09 years vs. 12.13 ± 0.18 years, *p *= 0.0006).

### Comparison of T1DM to NHANES 2001-2006

NHANES 2001-2006 data from female participants interviewed using the Reproductive Health Questionnaire and who were between 12 and 24 years of age (mean ± SE age = 18.2 ± 0.09) showed that the average age of menarche among adolescent females without diabetes (n = 3667) was 12.27 ± 0.038 years, which was significantly earlier than adolescent females with T1DM diagnosed prior to menarche (12.81 ± 0.09, *p *< 0.0001), and similar to adolescent females diagnosed after menarche (12.17 ± 0.19 years, *p *= 0.6993) (Figure 1). As the mean age of the NHANES cohort was older than the T1DM cohorts, we performed a linear regression analysis adjusting for current age, and found that the least square mean age of menarche among NHANES study participants at a current age of 15.8 was 12.27 ± 0.03, which remained significantly younger than the T1DM group diagnosed prior to menarche (12.81 ± 0.09, *p *< 0.0001), while the least square mean age of menarche among NHANES study participants at a current age of 16.5 was 12.28 ± 0.03, which was not significantly different from the T1DM cohort diagnosed after menarche (12.17 ± 0.19, *p *= 0.6922). A further analysis was conducted using only adolescent females who were at least 16 years of age (e.g. without primary amenorrhea), to ensure that differences in ascertainment of younger adolescents didn't explain the observed differences in age of menarche. The average age of menarche among adolescent females with T1DM diagnosed before menarche and age 16-24 at the visit (n = 101) was 13.0 ± 0.13 years, which was significantly older compared to the average age of menarche among female NHANES participants (n = 2272) of 12.5 ± 0.05 years (*p *= 0.001) and adolescent females with T1DM diagnosed after menarche (n = 30) of 12.32 ± 0.22 (*p *= 0.007).

### Age at menarche and clinical factors

A1c at menarche was available for 165 of 185 subjects with T1DM prior to menarche. A1c was not associated with age at menarche, although mean A1c (9.3%) was well above the recommended target for optimal diabetes control. Out of the 165 girls, 30 (18%) had an A1c at or below the target (A1c ≤ 7.5%) at the time of menarche. However, there was no difference in age of menarche between those who achieved the target A1c (12.61 ± 0.21) and those who had not (12.91 ± 0.10, *p = 0.20*). When those with menarche within one year of diagnosis with T1DM were eliminated from analysis in order to avoid bias due to the 'honeymoon' period, 21 (15%), of the remaining 136 adolescent females had A1c ≤ 7.5%. Although those adolescent females who reached the target A1c in this subset tended to have an earlier age of menarche (12.63 ± 0.21) than those who did not (12.99 ± 0.10), the difference did not reach statistical significance *(p *= 0.12).

There was a significant negative correlation between age of menarche and BMI z-score at menarche (r = -0.23, p = 0.0029). In multivariable linear regression adjusted for age at T1DM onset and A1c at menarche, BMI z-score remained significantly associated with age of menarche (β = -0.32, *p *= 0.005), as was age at T1DM onset (β = 0.06, *p *= 0.02), but not A1c (β = 0.08, *p *= 0.15)

### Race/ethnicity

**Figure 2:**
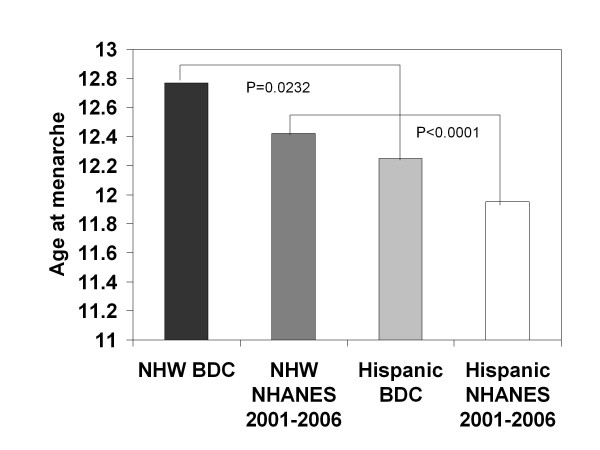
**Age of menarche in NHW and Hispanic patients**. Age of menarche in NHW BDC patients versus NHW NHANES sample (*P < 0.0001*) and Hispanic BDC patients versus Hispanic NHANES sample (*P *= 0.0653). NHW BDC versus Hispanic BDC (*P = 0.0232*).

Age at menarche by race/ethnicity for the BDC sample and NHANES data are shown in Figure 2. In the BDC sample, females of Hispanic origin (n = 34) reported earlier menarche than non-Hispanic white (NHW) (n = 185) females (12.25 ± 0.20 vs. 12.77 ± 0.09, *p *= 0.0232). In multivariable linear regression analysis adjusting for BMI z-score and A1c at menarche, this difference in age of menarche persisted for Hispanic vs. NHW adolescent females (least square means 12.33 ± 0.24 vs. 12.94 ± 0.09, *p *= 0.019)

In the NHANES sample, NHW (n = 1114) females without diabetes had an average age at menarche of 12.42 ± 0.05 years, which was significantly younger than the age of menarche in NHW girls in the BDC sample (*p *< 0.0001). In the NHANES sample, those of Hispanic origin (n = 1290) without diabetes had an average age of menarche of 11.95 ± 0.09 years, which was not significantly different from the age at menarche among Hispanic adolescent females in the BDC sample (*p = 0.0653*).

In the BDC sample, NHW girls with premenarchal presentation of T1DM (n = 151) had a later age of menarche than those who developed T1DM after menarche (n = 34) (12.92 ± 0.09 vs 12.32 ± 0.18, *p = *0.0062). Subjects in the BDC sample who were of Hispanic origin and who developed T1DM before menarche (n = 25) also had later menarche than those who developed T1DM after menarche (n = 7) (12.25 ± 0.23 vs. 11.91 ± 0.64, *p = 0.5461*), although this did not reach significance. NHW girls who had a premenarchal presentation of T1DM (n = 151) reported a later age of menarche than Hispanic girls who developed T1DM prior to menarche (n = 25) (12.92 ± 0.09 vs 12.25 ± 0.23, *p = 0.0083*).

### 21 Hydroxylase autoantibodies

21-Hydroxylase (OH) autoantibody titers were obtained through retrospective review of the chart. Results were available on 212 subjects and of those only 8 subjects had positive titers. The age of menarche in those negative for 21-OH antibodies (12.69 ± 1.20 years, *p = *0.4405) was not different from those with positive 21-OH antibodies (13.02 ± 0.85 years, *p = *0.4405). Since only 8 subjects had positive 21 OH antibodies the sample size did not allow for any further analysis.

### Severe hypoglycemia and/or episodes of DKA

There was no difference in age at menarche among girls with a history of at least one episode of DKA (n = 20) compared to girls without any DKA episodes (12.9 ± 1.1 vs. 12.8 ± 1.2 years of age, *p *= 0.78) or among girls with at least one episode of severe hypoglycemia (n = 11) compared to girls without any hypoglycemic episodes (12.7 ± 0.9 vs. 12.8 ± 1.2 years of age, *p = *0.72) occurring within the two years prior to the start of menarche. There was a trend towards more irregular menses among the adolescent females who had one or more episodes DKA before menarche compared to those with no episode of DKA (53% vs. 32%, *p *= 0.08)

### Menstrual regularity

Analysis of menstrual regularity was limited to adolescent females who were at least 2 years past menarche. Among the 181 adolescent females analyzed, 63 reported usually or always irregular cycles (35%) and the remaining 118 reported regular or very regular cycles. There was no difference in age of menarche, age of T1DM diagnosis, total physical activity, typical physical activity per/day, total insulin units kg/day, A1c, or BMI at the most recent visit between those who reported regular (always or usually) compared to irregular (always or usually) cycles. Total physical activity (OR = 0.973 [0.849-1.116], *p = *0.6949) and typical physical activity per/day (OR = 0.971 [0.845-1.116], *p = *0.6801) were not associated with irregular periods.

## Discussion

The present study is the largest to date to examine age at menarche and menstrual regularity among adolescents and young adults with TIDM. There is conflicting evidence as to whether the age at menarche in the general population has remained the same [33] or has declined [34]. The results from this study suggest that, despite improvements and advances in diabetes treatment and insulin regimens, the age of menarche in adolescent females with premenarchal presentation of T1DM continues to be delayed. Further, menstrual irregularity is common in youth with T1DM, affecting more than a third of the adolescent females studied.

We observed a negative correlation between age of menarche and BMI z-score, consistent with other studies which have also found that pubertal development [6] and menarche [8] tended to occur earlier in young women with T1DM whose BMI z-score was high than in those with a low BMI z-score. While this might suggest that poor metabolic control predicts later menarche, we found no correlation between age of menarche and A1c at menarche. Alternatively, the relationship between BMI z-score and menarche in young women with T1DM may mirror what has been shown in the general population [35].

To further explore glycemic control and age of menarche, we looked at episodes of DKA and severe hypoglycemia two years prior to starting menarche as measures of glycemic stability, and found that these factors were not associated with menarche delay, again pointing away from glycemic control as the explanation for delayed menarche.

Since the delay in menarche among adolescent females with T1DM has not significantly changed in the last several decades [13,15] despite improvements in diabetes management, this suggests that perhaps other factors besides glycemic control could be contributing to the menarchal delay in adolescents with TIDM. We examined whether the presence of 21 OH autoantibodies was associated with menarche delay and did not find a significant association, but since only a small number of subjects had positive 21 OH antibodies we are unable to draw any conclusions. To our knowledge there have been no other studies that have looked at the association of menarchal delay in adolescents with T1DM and positive 21-OH autoantibodies. It is possible that the presence of autoantibodies that have been linked to the menstrual cycle disturbances experienced in T1DM females [36] and premature ovarian failure in non-diabetic individuals [37-40] could be contributing to the delay in menarche. However, since positive 21-OH antibodies were not common in our study population, this mechanism is unlikely to explain the menarche delay we observed.

It is possible that the significant period of weight loss [41] and physiologic stress preceding the diagnosis of T1DM could contribute to the delay in menarche. In a study looking at 928 randomly selected adolescent schoolgirls aged 12-18 years with malnutrition it was found that menarche and start of puberty were delayed by approximately 1.5-2 years compared to a US reference population [42]. It is also known that chronic medical diseases other than TIDM, such as sickle cell disease [43] are associated with menarchal delay. Thus, it's possible that the initial weight loss period prior to diagnosis of T1DM, as well as the presence of chronically abnormal metabolism associated with T1DM could contribute to menarchal delay.

Approximately one third of young women with T1DM of reproductive age will suffer some form of menstrual dysfunction [44] and our results are consistent with this finding. In contrast, reported rates of menstrual irregularity in women without T1DM are 50% less than those with T1DM [15,45]. We found that there was no difference in age of menarche or A1c at study visit between those who had regular versus irregular menstrual periods. It is possible that other factors besides glycemic control are involved in menstrual regularity in women with T1DM. In a study looking at 53 T1DM girls, Snajderova found ovarian autoantibodies in 67.9% of the girls and speculated that the presence of these antibodies and other autoantibodies could somehow be linked to the menstrual cycle disturbances experienced in T1DM females (36). However, in our study we found that the presence of 21-OH autoantibodies was not increased in subjects with menstrual irregularity (Data not shown). However, even in patients with chronic specific organ specific autoimmune disease the presence of 21 OH antibodies is uncommon [45]. Thus, the young age of our study participants and small sample size the number of subjects with positive 21 OH antibodies were limited, making these results difficult to interpret. Others have speculated that the oligomenorrhea noted in T1DM is principally hypothalamic in origin and may represent intermittent (and perhaps reversible) failure of the GnRH pulse generator [46].

There were some limitations to our study. First, the participants were predominantly of NHW and Hispanic race/ethnicity, and so we were not able to provide reliable estimates of age of menarche in other racial/ethnic groups. Also, we did not have a non-diabetic control group from our population with whom to compare menstrual irregularity. In addition, there is also the potential for recall bias with the use of self reported questionnaires. However, the age of menarche has been shown to be reliable by recall, as it is usually a date that is well remembered [47]. Because the majority of our subjects were adolescent and relatively close to menarche, their recall may even be more reliable than in older women. The small sample size for some groups also limits the interpretation of negative findings, as it is likely that some of these findings could be explained by lack of power.

In conclusion, despite improved diabetes treatment in recent decades, a delay in menarche, although clinically modest, is still observed among girls with T1DM. Adolescent females who developed T1DM before menarche had a later onset of menarche than both adolescent females who developed T1DM after menarche and adolescent females those without diabetes from the NHANES 2001-2006 survey. This later age of menarche was not associated with A1c, even when comparing those with good control (A1C <7.5%) to those with less adequate control. Menstrual irregularity was common in our study participants, but was not associated with age of menarche, age of T1DM diagnosis, A1c, current BMI, physical activity or insulin dose. There are most likely other factors involved in the timing of menarche and menstrual regularity in girls with TIDM, and more research is needed in this area so that the etiology and consequences of delayed age at menarche in this population can be better understood.

## Competing interests

The authors declare that they have no competing interests.

## Authors' contributions

Both GJK and JS contributed to the discussion and revisions and edits of the manuscript. KM contributed to data analysis and design of table and figures. All three are employed by the University of Colorado and work for the Barbara Davis Center for Childhood Diabetes. RR contributed to patient recruitment. BS developed the research design, recruited subjects, researched data, wrote manuscript, contributed discussion and revised and edited the manuscript. There was no other editorial assistance of a colleague in the preparation of the manuscript. All authors have read and approved the final manuscript.
